# An *In Vitro* Anticancer Activity Evaluation of *Neolamarckia cadamba* (Roxb.) Bosser Leaves’ Extract and its Metabolite Profile

**DOI:** 10.3389/fphar.2021.741683

**Published:** 2021-10-13

**Authors:** Shakirah Razali, Al’aina Yuhainis Firus Khan, Alfi Khatib, Qamar Uddin Ahmed, Ridhwan Abdul Wahab, Zainul Amiruddin Zakaria

**Affiliations:** ^1^ Department of Biomedical Science, Kulliyyah of Allied Health Sciences, International Islamic University Malaysia, Kuantan, Malaysia; ^2^ Pharmacognosy Research Group, Department of Pharmaceutical Chemistry, Kulliyyah of Pharmacy, International Islamic University Malaysia, Kuantan, Malaysia; ^3^ Faculty of Pharmacy, Airlangga University, Surabaya, Indonesia; ^4^ Department of Biomedical Sciences, Faculty of Medicine and Health Sciences, Universiti Malaysia Sabah, Kota Kinabalu, Malaysia; ^5^ Laboratory of Halal Science Research, Halal Products Research Institute, Universiti Putra Malaysia, Serdang, Malaysia

**Keywords:** *Neolamarckia cadamba*, breast cancer, gas chromatography, gene expression assay, mechanisms of action, *in vitro* anticancer

## Abstract

The leaves of *Neolamarckia cadamba* (NC) (Roxb.) Bosser (family: Rubiaceae) are traditionally used to treat breast cancer in Malaysia; however, this traditional claim is yet to be scientifically verified. Hence, this study was aimed to evaluate the anticancer effect of NC leaves’ ethanol extract against breast cancer cell line (MCF-7 cells) using an *in vitro* cell viability, cytotoxicity, and gene expression assays followed by the gas chromatography analysis to further confirm active principles. Results revealed 0.2 mg/ml as the half maximal inhibitory concentration (IC_50_) against MCF-7. The extract exerted anticancer effect against MCF-7 cells in a dose- and time-dependent manner. The cell cycle assay showed that the extract arrested MCF-7 cells in the G0/G1 phase, and apoptosis was observed after 72 h by the Annexin-V assay. The gene expression assay revealed that the cell cycle arrest was associated with the downregulation of *CDK2* and subsequent upregulation of p21 and cyclin E. The extract induced apoptosis *via* the mediation of the mitochondrial cell death pathways. A chromatography analysis revealed the contribution of D-pinitol and myo-inositol as the two major bioactive compounds to the activity observed. Overall, the study demonstrated that NC leaves’ ethanol extract exerts anticancer effect against MCF-7 human breast cancer cells through the induction of apoptosis and cell cycle arrest, thereby justifying its traditional use for the treatment of breast cancer in Malaysia.

## Introduction

Breast cancer is the most commonly occurring cancer in women and the second most common cancer overall in terms of fatality after lung cancer (Bray et al., 2018). The rate of breast cancer patients’ cases is increasing every year, both in developed and developing countries (Torre et al., 2015). It is estimated that 26 million new cancer cases and 17 million cancer deaths are likely to occur per year globally by 2030 (Thun et al., 2010). In Asia alone, the incident from 2008 to 2030 is estimated to increase from 6.1 to 10.7 million cancer cases and 4.1 to 7.5 million deaths (Sankaranarayanan et al., 2014). Malaysia is one of the Asian countries with an increased prevalence of breast cancer cases (Azizah et al., 2016), affecting both genders but increased exponentially with age in women and thus accounted for the leading cause of cancer-related deaths among women in Malaysia (Lukong, 2017). Surgery, radiotherapy, chemotherapy, hormonal therapy, and gene-targeted therapy have been greatly used in treating breast cancer patients. The advances of current treatments and detecting methods have contributed to the increased survival rate (Blumen et al., 2016). Unfortunately, these treatments cause short-term and long-term side effects for the patients. Moreover, studies have demonstrated that the burden of the cost related to cancer treatment is uncharacteristically rising (Blumen et al., 2016; Trogdon et al., 2017). Therefore, using natural sources as alternative, effective, and non-invasive entities to treat cancer is warranted.

According to the World Health Organization (WHO), many countries, including developing countries, still use plants and natural source–related products for therapeutic purposes (Rayan et al., 2017). About 60% of anticancer agents have been originated from the natural sources globally (Newman and Cragg, 2016). The nature-derived compounds are readily available, generally more tolerated, and considered non-toxic to normal human cells (Shah et al., 2013).

*Neolamarckia cadamba* (Roxb.) Bosser (family: Rubiaceae) is a tree that is traditionally used for the treatment of various illnesses. This underexplored evergreen tropical plant has been widely used in the Ayurvedic medicine system of India in treating eye infection, skin diseases, dyspepsia, gum-related troubles, stomatitis, cough, fever, anemia, blood disorders, and stomach ache (Dubey et al., 2011; Pandey and Negi, 2016). In addition, the leaves of this plant have been shown to possess several pharmacological properties, including antioxidant (Kaur and Kumar, 2011; Chandel et al., 2012), antidiabetic (Ahmed et al., 2011), antitumor (Dolai et al., 2012), anti-inflammatory, antipyretic, analgesic (Mondal et al., 2009), antimicrobial (Rafshanjani et al., 2014), and anticancer effects (Singh et al., 2013). Furthermore, the *N. cadamba* leaves have also been used as a topical application to treat breast cancer. However, this traditional application has never been scientifically investigated to verify its anticancer effect. Therefore, the main purpose of the study was to evaluate the anticancer effects of *N. cadamba* leaves’ extract on breast cancer cell line (MCF-7) with emphasis on the mechanism of action, that is, apoptosis induction and cell cycle arrest. The characterization of *N. cadamba* leaves’ extract was also carried out using a gas chromatography–mass spectrometry (GCMS) approach to identify bioactive compounds.

### Novelty

1. It is the pioneer study highlighting the anticancer effect of *Neolamarckia cadamba* (Roxb.) Bosser leaves’ ethanol extract on breast cancer cells for the first time.
2. *Neolamarckia cadamba* leaves’ ethanol extract showed dose- and time-dependent anticancer effects.
3. Treated cells were arrested in the G1 phase and underwent apoptosis *via* the mitochondria pathway.
4. Findings of this study further support the traditional use of *Neolamarckia cadamba* (Roxb.) Bosser leaves as an anticancer agent in the treatment of breast cancer.

## Materials and Methods

### Materials and Chemicals

The GCMS reagents were purchased from Sigma-Aldrich, United States (Pyridine and N-Methyl-N-(trimethylsilyl)tri-fluoroacetamide-MSTFA) and Supelco, United States (methoxyamine HCl). The normal human fibroblast (HDF) and human breast adenocarcinoma (MCF-7) cells were obtained from American Type Culture Collection (ATCC), United States. All cells were maintained in complete growth medium (CGM) consisting of Dulbecco’s modified Eagle medium (DMEM) (Nacalei Tesque, Japan), 10% fetal bovine serum (Nacalei Tesque, Japan), and 1% of penicillin–streptomycin (Nacalei Tesque, Japan). The phosphate-buffered saline (PBS) from Gibco, United States, was used for cell-washing purposes. Reagents for apoptosis (Nexin reagent) and a cell cycle reagent were purchased from Merck Millipore, United States. Kits used for the mRNA analysis were purchased from Analytik Jena, Germany (InnuPREP DNA/RNA mini kit), BIOLINE, United Kingdom (SensiFAST cDNA synthesis and SensiFAST SYBR® No-ROX kits), and Integrated DNA technologies, Singapore (primer).

### Preparation of *Neolamarckia cadamba* Leaves’ 80% Ethanol Extract (NC)

Fresh leaves of *N. cadamba* were collected from Balok, 25200 Kuantan, Pahang DM, Malaysia, and deposited (voucher specimen #: PIIUM 0266) in the Herbarium, Kulliyyah of Pharmacy, IIUM, Malaysia. The leaves were prepared according to the previous method (Liu et al., 2017) with slight modification. The leaves were washed and air-dried. The dried leaves were pulverized and soaked in 80% ethanol three times using an ultrasonic sonicator. The extract was filtered, concentrated by a rotary evaporator, and freeze-dried. It was stored at −20°C until further usage. The extract was dissolved in 80% ethanol to obtain a stock solution of 100 mg/ml. Working solutions were prepared fresh from ethanol stock in the DMEM medium by serial dilutions. The solutions were then filtered with a sterilized 0.22 µl syringe filter before being used in an *in vitro* assay.

### Gas Chromatography–Mass Spectrometry (GCMS) Analysis of NC

Derivatization of the samples was performed for the GCMS analysis following the method described (Liu et al., 2017). Briefly, about 50 µl pyridine, 100 µl of methoxyamine HCl (20 mg/ml in pyridine), and 300 µl of MSTFA were added to the extract. The extract solutions were filtered and covered with aluminum foil to be left overnight at room temperature before the analysis. The GCMS analysis was carried out using GCMS-TQ8040 (Shimadzu, Japan), where the analysis was carried out using a DB-5MS 5% phenyl methyl siloxane (30 mm × 0.25 mm, i.d. × 0.25 µm thickness). The initial oven temperature used was set to 50°C for 5 min and raised to 200°C at the rate of 5°C/min and held for 5 min before reaching the target temperature of 300°C at the same rate. The carrier gas applied was helium with a flow rate of 1.0 ml/min, while the injector and detector temperature were set up to 250 and 280°C, respectively. Full-scan mass spectra were retrieved by setting parameters ranging from 50 to 550 *m*/*z*. The chromatograms of the compounds were compared to the database of the National Institute of Standards and Technology (NIST) 2014.

### Effect of NC on Cell Viability of MCF-7 and HDF Cell Growth

All cells were maintained in a sterilized environment set to 37°C with 5% CO_2_ humidified atmosphere. The cell viability of NC-treated cells was measured using the trypan blue exclusion (TBE) method as previously described (Hsieh et al., 2005). In determining the median concentration of NC on MCF-7 cells, the cells were seeded at 4.7 × 10^4^ cells/ml in 6-well plates. After overnight, different concentrations of NC prepared by serial dilution (500.0, 250.0, 125.0, 62.5, 31.3, 15.6, 7.8, 3.9, 1.95, and 0.9 μg/ml) were added to MCF-7 cells and kept for 72 h before performing cell density evaluation using TBE. MCF-7 cells were exposed to NC for 24, 48, 72, and 96 h prior evaluation with the TBE assay to determine the MCF-7 growth exposure with NC. The cytotoxicity of NC against normal HDF cells, which were cultured at 5.0 × 10^4^ cells/ml in 6-well plates, was determined by examining the IC_50_ value of NC after 72 h. The untreated cells were not exposed to NC and were used as a negative control.

### Cell Cycle Analysis

MCF-7 cells collected from the well were prepared in triplicate prior to staining with the cell cycle reagent (Guava Cell Cycle Reagent), following the manufacturer’s instructions. The PI-stained cells were observed using a Guava EasyCyte flow cytometer system (Hsieh et al., 2005).

### Apoptosis Analysis Using Annexin V

Briefly, MCF-7 cells were exposed to NC for 72 h. The collected cells were treated with the FITC Annexin-V Apoptosis Detection Kit I, following the manufacturer’s instructions. The cells were treated with 1x binding buffer, FITC Annexin-V, and propidium iodide (PI) before their application to the Guava easyCyte flow cytometer system (EMD Millipore, Germany) (Hsieh et al., 2005).

### Real-Time Quantitative PCR Analysis of Target Genes

The quantification of the mRNA expression of the targeted primer mRNA corresponding to apoptosis, cell cycle, and metastasis was carried out following our previously published study (Strober, 2015). Briefly, the RNA extraction kit was used to extract the total RNA from the plant treated and non-treated MCF-7 cells after 72 h. The RNA’s purity and integrity were evaluated prior to complementary DNA (cDNA) synthesis. The cDNA was prepared using a cDNA synthesis kit with 200 ng of RNA template following the manufacturer’s instructions. Primers were selected from the National Centre for Biotechnology Information (NCBI) database based on the criteria listed below.

i. Melting temperature, Tm (59–65°C)
ii. Amplicon size (70–150 bases)
iii. Forward and reverse primers (spanning exon–exon junctions)
iv. Primer’s 3′ end with a C or G residue
v. GC content (40–60%)
vi. Primer’s sequence (18–25 nucleotides)

The PCR amplifications were conducted using SYBR green in CFX96 Touch™ Real-Time PCR (Bio-Rad, United States). The efficiency of the primer PCR was evaluated using 1:10 serial dilutions of the cDNA sample. The standard curve’s amplification efficiency (E) range was set between 93.5 and 115.4%, while the correlation coefficients (R2) ranged from 0.984 to 0.998. The optimized parameters of the amplifications include 2 min at 50°C, 10 min at 95°C, 45 cycles of 15 s at 95°C, and 1 min at 60°C. The CFX Manager Software was used to analyze the results obtained by applying threshold cycle (CT) values. The reference genes used were glyceraldehyde 3-phosphate dehydrogenase (GAPDH) and β-actin (ACTB). The primers’ detailed information is displayed in Supplementary Table S1.

### Statistical Analysis

Statistical and the IC_50_ values’ (half maximal inhibitory concentration) calculations were carried out using GraphPad Prism software version 6. The cytotoxicity data were analyzed using multiple *t*-tests. The flow cytometry analysis and gene expression data were determined using multiple *t*-tests (Holm–Sidak method). All data were displayed as mean ± SD (standard deviation). * Signifies the significant difference when *p* < 0.05.

## Results

### Gas Chromatography–Mass Spectrometry Analysis of NC

Table 1 presents the putative compounds detected in the NC leaves’ 80% ethanol extract. The metabolites with the similarity index (SI) of more than 85% based on the National Institute of Standards and Technology (NIST) 14 database can be regarded as the putative compounds. The chromatogram showed the highest peak area corresponding to D-pinitol, followed by myo-inositol, oleic acid, hexadecanoic acid, and octadecanoic acid.

**TABLE 1:** Putative compounds in NC leaves’ extract identified by GCMS.

No.	Retention time	Name of compound	Area %	Similarity index
1	14.25	Phenol, 2,4-bis(1,1-dimethylethyl)-	0.21	93
2	16.08	Levoglucosan	0.23	91
3	20.36	β-D-(+)-Talopyranose	0.38	92
4	20.78	D-pinitol	14.18	86
5	20.98	Hexadecanoic acid	0.52	91
6	22.13	Myo-inositol	7.98	90
7	23.22	Oleic acid	0.66	89
8	23.48	Octadecanoic acid	0.43	90

### Breast Cancer Cells (MCF-7) Viability After NC Treatment

**FIGURE 1:**
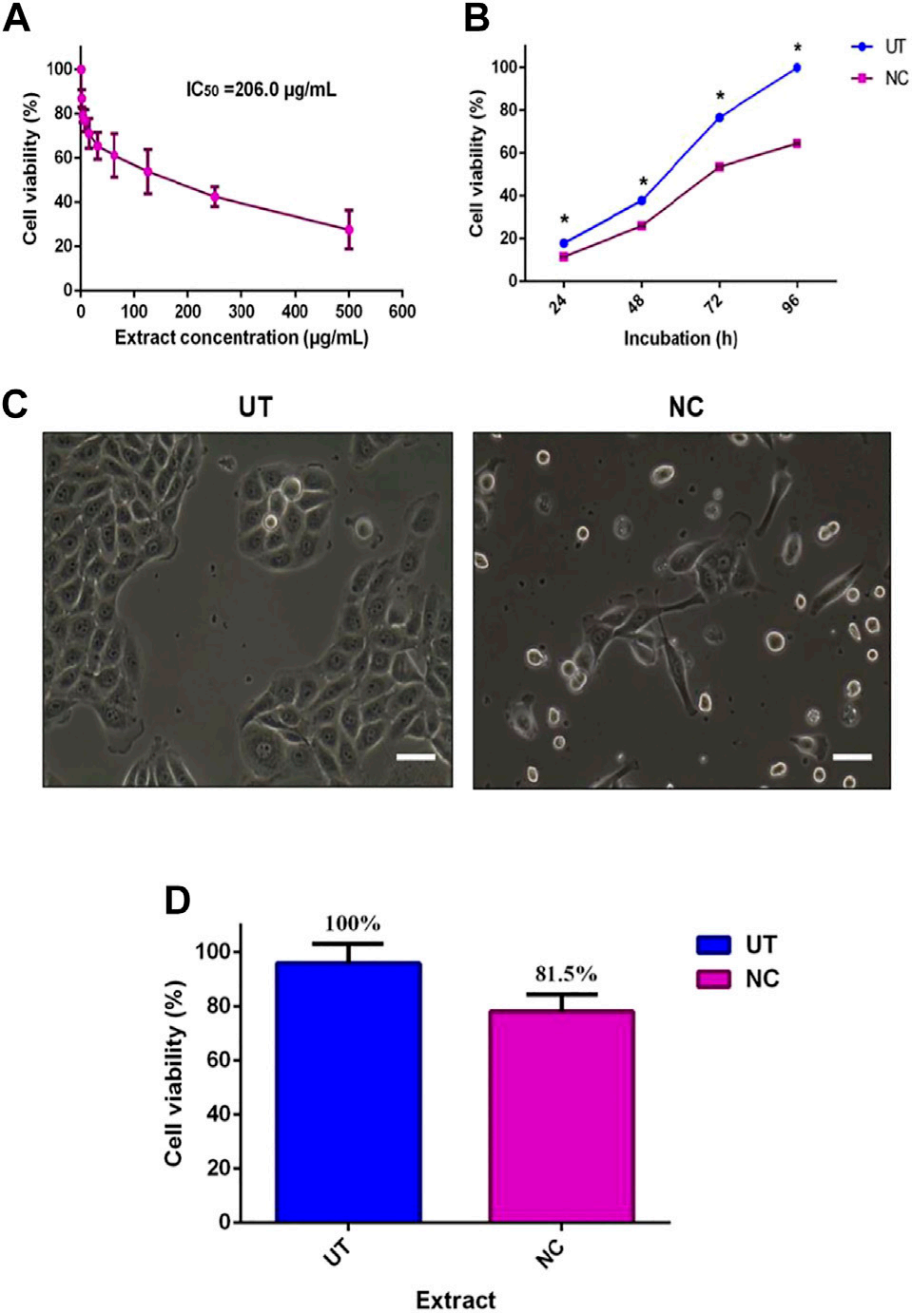
Effect of NC leaves’ 80% ethanol extract on MCF-7 cell viability. **(A)** The median concentration of MCF-7 treated with NC leaves’ 80% ethanol extract for 72 h. **(B)** Antiproliferative effect of NC leaves’ 80% ethanol extract at 24, 28, 72, and 96 h with untreated cells (UT) as controls. **(C)** Image of MCF-7 cells with NC leaves’ 80% ethanol extract treated and untreated at 72 h. **(D)** Cytotoxic effect of NC leaves’ 80% ethanol extract median concentration on normal human dermal fibroblast (HDF) cells at 72 h incubation. The results presented as mean ± SD (*n* = 3) (bar graph = 25 μm). * indicates *p* < 0.05.

The inhibitory activity of NC leaves’ 80% ethanol extract was investigated against MCF-7 cells. The cell viability was directly tested using the trypan blue exclusion assay method where the dead cells were stained blue. Figure 1A shows the antiproliferative activity of NC leaves’ 80% ethanol extract on MCF-7 cells after 72 h of incubation. The finding revealed an IC_50_ of 206.0 ± 3.4 μg/ml. Cell viability was found to decrease with the increasing concentration of the extract used. Figure 1B demonstrates the IC_50_ value of NC to inhibit MCF-7 growth with cell viability percentage difference between NC and UT by 6.3, 12.2, 23.1, and 35.4% at 24, 48, 72, and 96 h, respectively. From the findings, it can be seen that the NC inhibition effect on MCF-7 growth increased over time. Figure 1C depicts the image of MCF-7 cells treated with NC in comparison to the untreated as a control. The image shows the cells exhibiting a distinguished plasma membrane with intact nuclei, and cells were seen to grow adjacent to the neighboring cells in a monolayer. Most of the cells were attached to the tissue culture plate. In contrast, cells treated with NC extract for 72 h showed apoptotic-like features including cell rounding, loss of contact with the adjacent cells, cell shrinkage, and formation of apoptotic bodies. Figure 1D presents the viability of normal HDF cells exposed to NC leaves’ 80% ethanol extract for 72 h. The graph reveals that NC leaves’ 80% ethanol extract had slight toxicity on HDF cells by inhibiting cell growth by 19.5% when compared to UT. Overall, the NC ethanol extract exhibited a concentration and time inhibition pattern related to the MCF-7 cell growth and slight toxicity on normal HDF cells. IC_50_ obtained was used for further investigation in this study.

**GRAPHICAL ABSTRACT:**
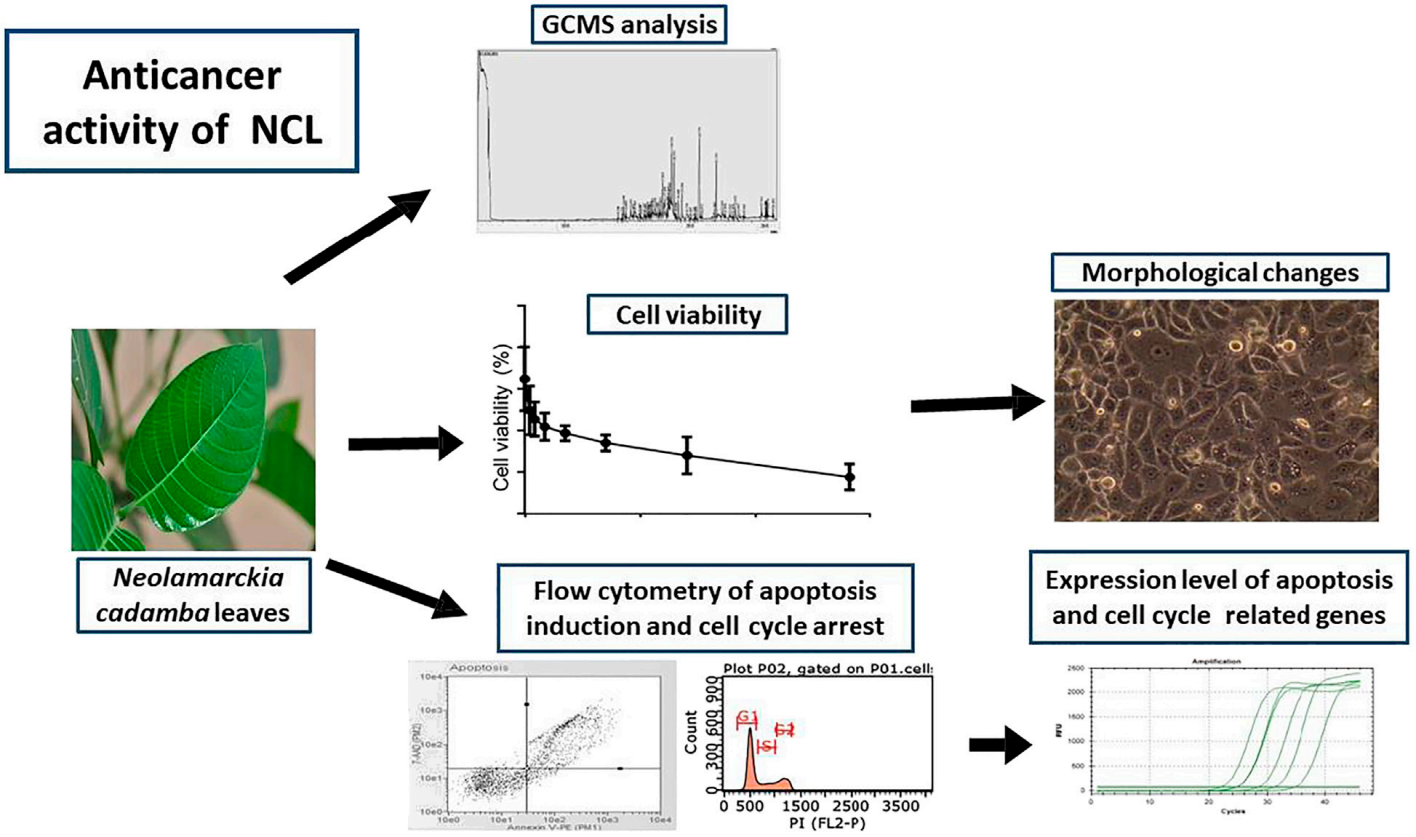


### Cell Cycle Analysis of NC Against MCF-7 Cells

**FIGURE 2:**
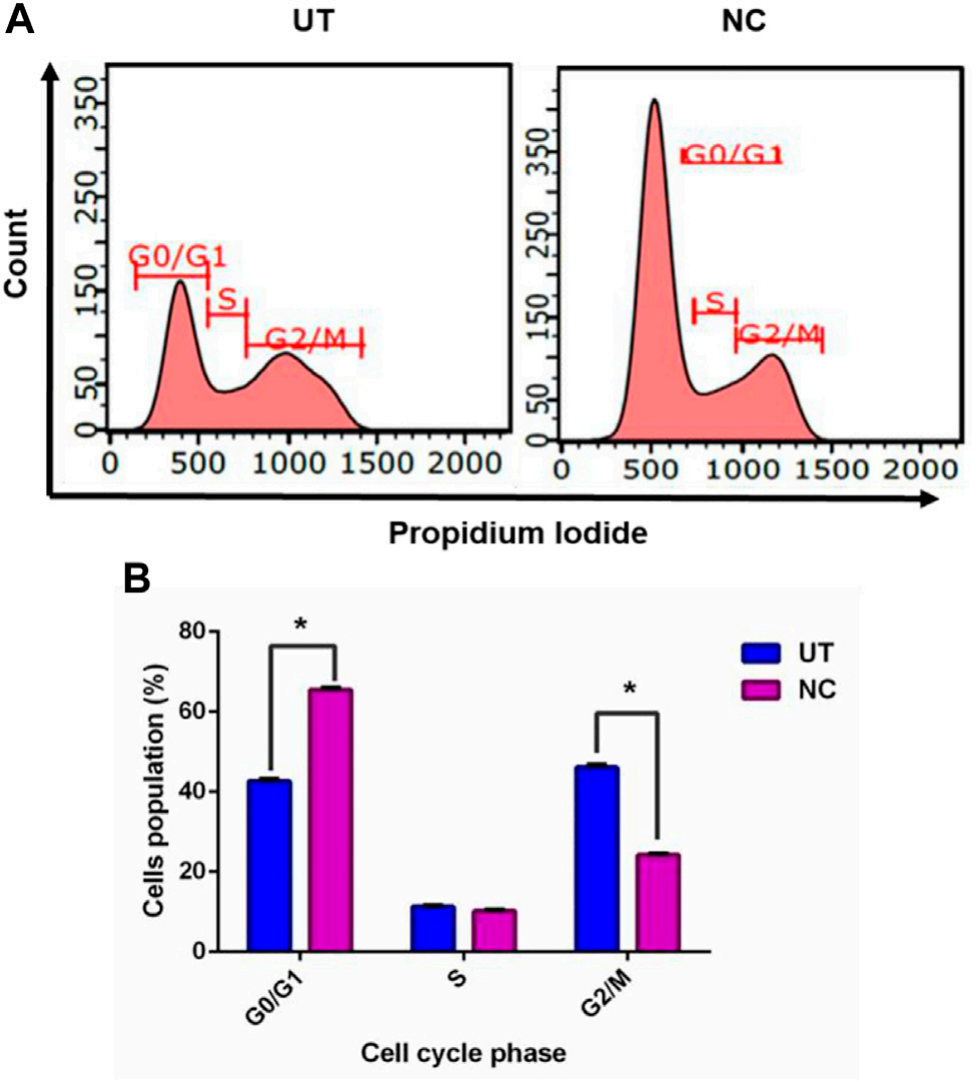
Effect of NC leaves’ 80% ethanol extract on the MCF-7 cell cycle. **(A)** Representation of DNA histogram of NC leaves’ 80% ethanol extract–treated cells, with UT as control at 72 h of incubation. DNA histograms display cell cycle phases of treated cells, namely, G1/G0, S, and G2/M (*n* = 3). **(B)** The proportion of cells according to the cell cycle phase are presented in percentage mean ± SD (*n* = 3). * indicates *p* < 0.05 concerning untreated.

To understand the growth inhibition found earlier, NC leaves’ 80% ethanol extract–treated MCF-7 cells were subjected to cell cycle analysis with UT serving as control. Figure 2A demonstrates MCF-7 cells treated with NC leaves’ 80% ethanol extract and UT DNA histogram at 72 h of incubation, which indicates that NC leaves’ 80% ethanol extract exerted MCF-7 arrest in G1/G0 compared to UT. Figure 2B shows the proportion of cells according to cell cycle phase from Figure 2A. The graph reveals that NC leaves’ 80% ethanol extract significantly induced G1/G0 arrest in MCF-7 cells evidenced by the increase of cell population by 22.9% in G1/G0 and simultaneous decrease of G2/M by 21.9% compared to UT.

### Apoptosis Analysis of NC Leaves 80% Ethanol Extract Against MCF-7 Cells

**FIGURE 3:**
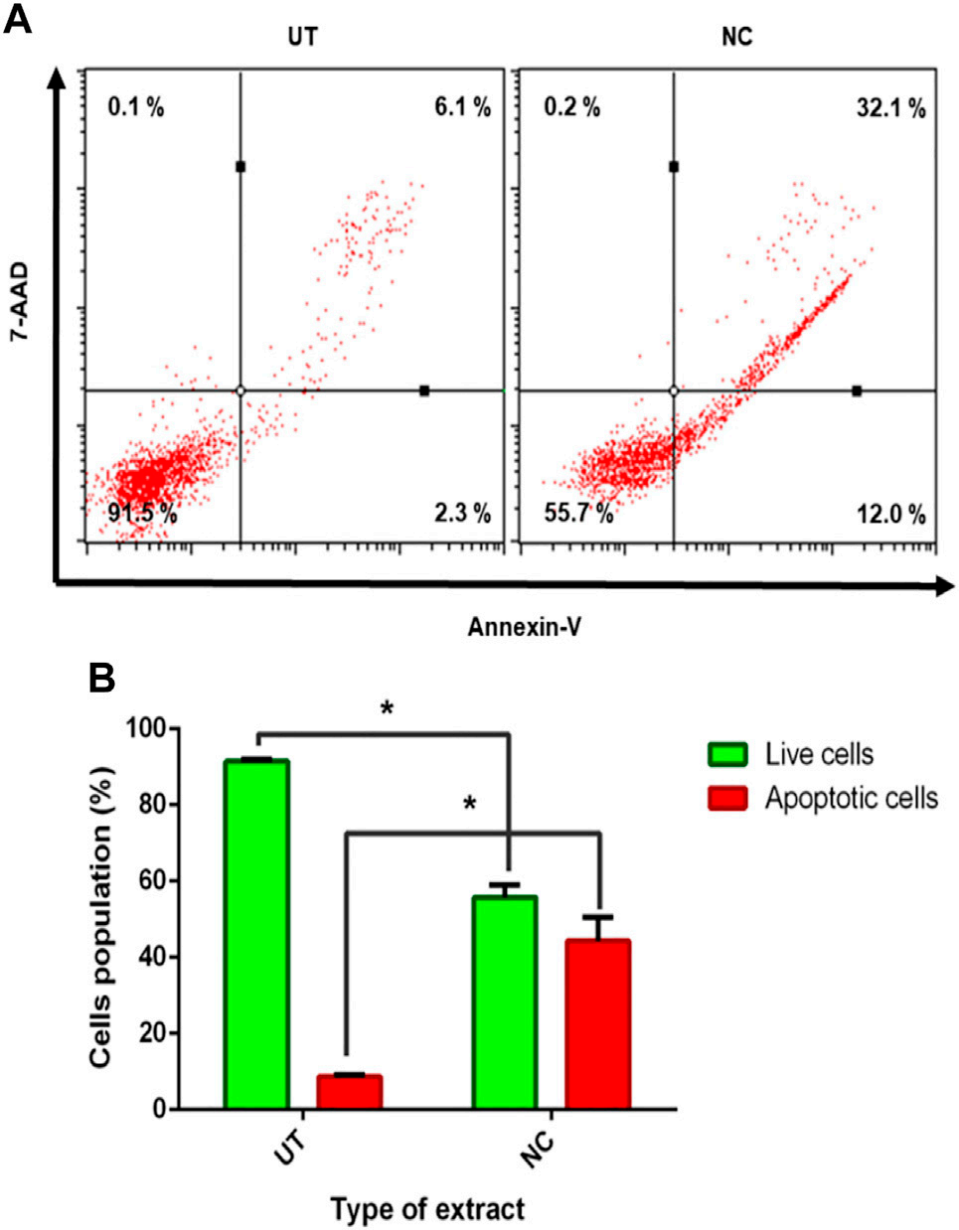
Effect of NC leaves 80% ethanol extract on MCF-7 cells. **(A)** Representation of dot plot distribution of cells in living, death, and early and late apoptosis quadrants of MCF-7 cells treated with NC leaves 80% ethanol extract at 72 h with UT as control (*n* = 3). **(B)** The proportion percentages of apoptotic (early and late apoptosis) and live cells are presented as percentage mean ± SD (*n* = 3). * indicates *p* < 0.05 concerning untreated.

As evidenced earlier, NC leaves’ 80% ethanol extract showed growth inhibition and cell death; hence, further investigation was conducted to evaluate the possibility of cells to undergo apoptosis using flow cytometer analysis. The Annexin-V assay helped to differentiate individual cells in live, early apoptotic, late apoptotic, and necrotic quadrants following 72 h of NC leaves’ 80% ethanol extract treatment to MCF-7 cells. Figure 3A shows MCF-7 cells, NC leaves’ 80% ethanol extract-treated cells, and UT dot plot distribution in quadrants. The Annexin-V assay revealed that NC leaves’ 80% ethanol extract–treated cells induces early and late apoptosis by 12.0 and 32.1% compared to UT. On the contrary, UT cells are majorly located in the live quadrant. Figure 3B demonstrates the proportion percentages of apoptotic (early and late apoptosis) and live cells of NC leaves’ 80% ethanol extract–treated and UT MCF-7 cells. The proportion of apoptotic percentage from NC leaves’ 80% of ethanol extract–treated cells was significantly higher by 44.3% compared to UT 8.6%, while the proportion of live cells percentage was higher in UT MCF-7 by 91.5% than in NC-treated cells by 65.7%.

### Effects of NC Leaves’ 80% Ethanol Extract on Gene Expression of MCF-7

**FIGURE 4:**
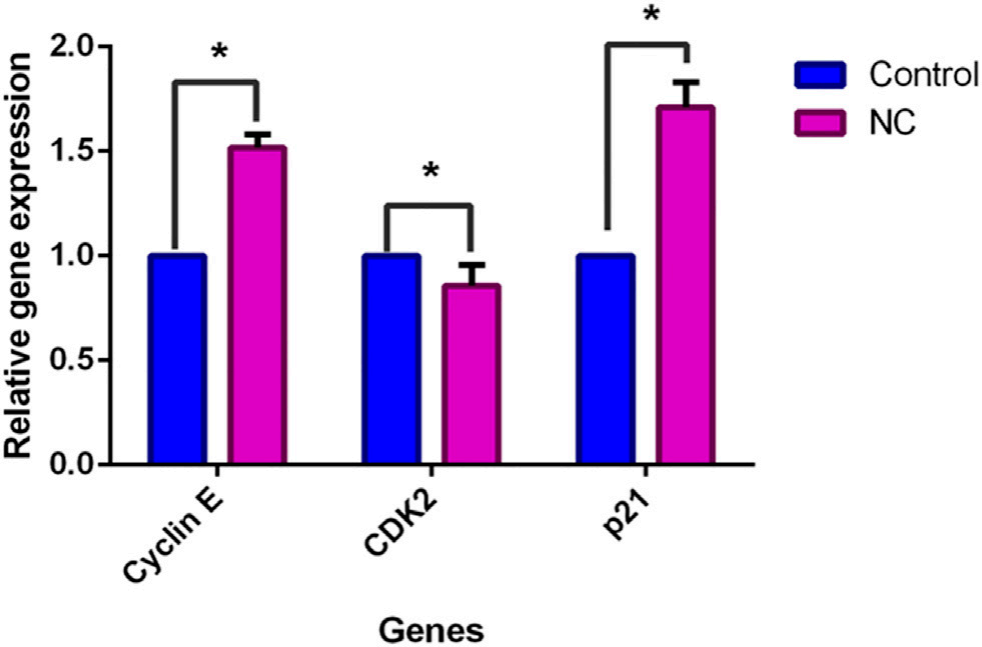
Effect of NC leaves 80% ethanol extract on cell cycle-related gene expression upon treatment with IC_50_ for 72 h. Relative expression for cyclin E, CDK2, and p21 were calculated by measuring the fold change with GAPDH and β-actin as reference genes in untreated cells (the ratio was equal to 1). Data presented in mean ± standard deviation of triplicates of the experiment. * indicates a significant difference when *p* < 0.05.

A deeper investigation was further performed to investigate gene expression of NC leaves’ 80% ethanol extract supplement to MCF-7 cells with regards to cell cycle arrest and apoptosis induction. Five genes related to apoptosis were measured for their expressions through the qPCR assay *viz*. Bax, Bcl-2, caspase-9, caspase-7, and cytochrome c. The expression of cell cycle–related genes on MCF-7–treated cells with a growth arrest at the G0/G1 phase, cyclin E, CDK2, and p21 were studied. Figure 4 shows that upon NC leaves’ 80% ethanol extract treatment, the expression of cyclin *E* and *p21* were found to be upregulated with 1.6 ± 0.54 and 1.7 ± 0.02 folds, respectively. Meanwhile, CDK2 was downregulated with 0.9 ± 0.07 folds. From this result, the upregulated expression of *p21* in NC-treated cells indicates that the *p21* inhibits the activity of *CDK2* and cyclin E catalytic complex formation which blocks the progression to the next stage of the cell cycle.

**FIGURE 5:**
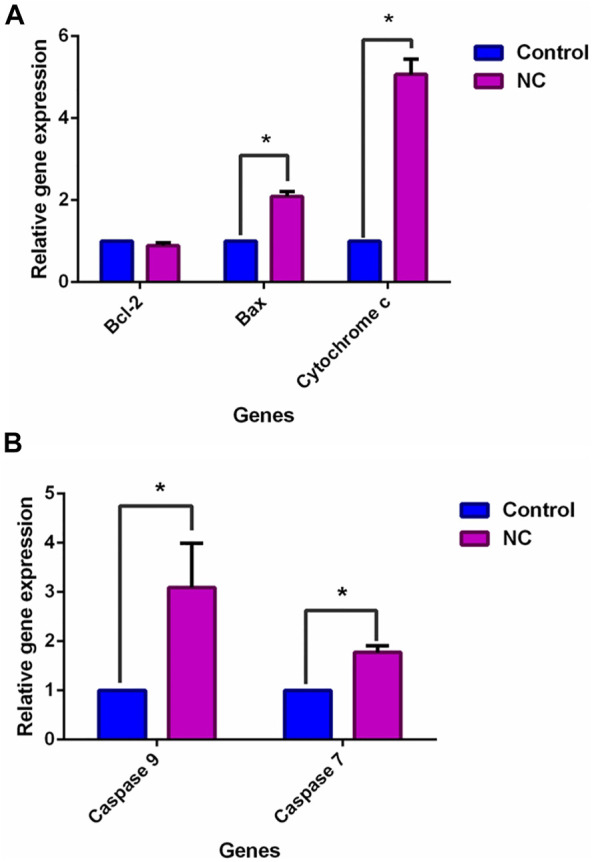
Effect of NC leaves 80% ethanol extract on apoptosis-related gene expression upon treatment with IC_50_ for 72 h. Relative expression for **(A)** Bcl-2, Bax, and cytochrome c, and **(B)** caspase-9 and caspase-7 were calculated by measuring the fold change with GAPDH and β-actin as a reference gene in untreated cells, whose ratio was equal to 1. Data presented in mean ± standard deviation of triplicates of the experiment. * indicates a significant difference when *p* < 0.05.

In Figure 5A, Bax (proapoptotic) and cytochrome c were downregulated by NC leaves’ 80% ethanol extract by 2.1 ± 0.07-fold and 5.1 ± 0.42-fold, respectively, whereas Bcl-2 was downregulated by 0.9 ± 0.04-fold. Meanwhile, in Figure 5B caspase family (caspase-9 and caspase-7) expressions were upregulated with NC leaves’ 80% ethanol extract treatment by 3.1 ± 0.65 and 1.8 ± 0.19-fold, respectively. The upregulation of Bax, cytochrome c, and caspases family corresponded with the downregulation of *Bcl-2* gene, suggesting that NC extract treatment lowered cell survival and induced apoptosis *via* the intrinsic mitochondrial pathway.

## Discussion

Throughout the whole world, breast cancer is affecting women the most compared to other types of cancers with a high incidence and mortality rate (Harbeck et al., 2019; Lin et al., 2019). Additionally, there are some undocumented and unverified claims for *N. cadamba* leaves that it is used to treat breast cancer in different parts of Malaysia; however, no scientific study using breast cancer cell lines has ever been carried out to confirm its potential in the management of breast cancer. Hence, this study was performed to verify *N. cadamba* leaves’ extract as a potential anticancer agent with minimal toxicities. In this study, NC leaves’ 80% ethanol extract was initially characterized with GCMS and then assessed for its *in vitro* anticancer properties and mechanism of action using MCF-7 cells. Phytochemical characterization of NC leaves s 80% ethanol extract through the GCMS analysis identified eight putative bioactive compounds including D-pinitol, myo-inositol, oleic acid, hexadecanoic acid, octadecanoic acid, β-D-(+)-talopyranose, levoglucosan, and phenol, 2, 4-bis(1,1-dimethylethyl).

Among the compounds, D-pinitol (6-methoxycyclohexane-1,2,3,4,5-pentol) was found to be the most abundant one. D-pinitol is a natural, sugar-like cyclitol, a cyclic polyol found in a variety of plants including soybean and has been reported to exert diverse biological activities particularly antioxidant, antiviral, antidiabetic, anti-inflammatory, and anticancer. D-pinitol has been reported to inhibit prostate cancer metastasis through the inhibition of αVβ3 integrin by modulating FAK, c-Src, and NF-κB pathways (Lin et al., 2013). Furthermore, a study revealed that d-pinitol was able to induce apoptosis in MCF-7 by upregulating Bax and downregulating Bcl2 expressions. It was also demonstrated that D-pinitol promoted apoptosis in MCF-7 cells *via* induction of p53 and Bax and inhibition of Bcl-2 and NF-κB (Rengarajan et al., 2014). Myo-inositol, the next most abundant bioactive compound in *N. cadamba* leaves’ extract, has been earlier isolated from fresh samples of *Cosmos caudatus* Kunth (Javadi et al., 2015) and *Sapindus mukorossi* Gaertn (Liu et al., 2018). Myo-inositol has been currently used clinically to treat polycystic ovary syndrome (PCOS) (Artini et al., 2013).

Hexadecanoic acid was identified in our study as another bioactive compound, and identified earlier from methanol (44.88%) and hexane (17.96%) extracts of *N. cadamba* leaves. Additionally, octadecanoic acid was also found in methanol, ethyl acetate, chloroform, and hexane extract (Zayed et al., 2014). N-hexadecanoic acid found in *Kigelia africana* subsp. africana (syn. *Kigelia pinnata*) leaves had caused cytotoxicity in HCT-116 cell lines (Ravi and Krishnan, 2017). Moreover, octadecanoic acid has induced apoptosis in breast cancer cells (Evans et al., 2009). Oleic acid found in almond oil (*Prunus dulcis* (Mill.) D. A.Webb) has displayed antiproliferative and anticancer effects on colon carcinoma cells (Mericli et al., 2017). Meanwhile, the ability of phenol, 2,4-bis(1,1-dimethylethyl) compound docked with Bcl-2 showing low binding affinity (−7.4 kcal/mol) after caryophyllene compound (−7.5 kcal/mol) indicated the potential of *Solanum trilobatum* L. extract compounds as a potent anticancer agent (Xavier et al., 2013).

The results showed that the population of MCF-7 cell lines was reduced to 50% from 100% of viable cells. NC extract exhibited a growth inhibitory effect toward MCF-7 in a concentration- and time-dependent manner in which the percentage of viable cells decreased in correspondence with the increase of NC extract concentration. Different bioactive compounds were reported in *N. cadamba* leaves using different solvents and associated with various biological activities, including antioxidant, anticancer, or antitumor activity (Zayed et al., 2014). Sun and Hai Liu (2006) described that the existence of different components in the natural sources may produce additive and synergistic effects rather than a single effect. The biochemical and different morphological changes such as cell shrinkage, cell blebbing, the formation of apoptotic bodies, nuclear condensation, and DNA fragmentation are the hallmarks of apoptosis (Ouyang et al., 2012). The apoptotic-like features of MCF-7 cells were observed under a phase-contrast inverted microscope. Cell rounding, cell shrinkage, membrane blebs, loose contact with adjacent cells, the formation of apoptotic bodies, and cell vacuolization were seen in the treated MCF-7 cells. The appearance of apoptotic cell features has also been reported in MCF-7 cells treated with rapamycin under inverted microscope observation (Khairi et al., 2014). Alteration in the morphology of MCF-7 cells following the NC extract treatment indicated that the cells experienced apoptosis (Wen et al., 2017). One of the main challenges with the current treatments is the capacity to hit cancer cells without killing normal cells surrounding the cancer cells. However, the findings in this study revealed that NC extract was slightly toxic toward normal cells.

Inhibition of proliferation and/or induction of apoptosis in cancer cells is considered one of the main important criteria for many anticancer agents (Barhoi et al., 2021; Orabi et al., 2021). The flow cytometric analysis in the present study revealed that MCF-7 cells underwent apoptosis upon treatment with NC, abundantly in late apoptosis. A similar finding was found on MCF-7 cells after the treatment with rapamycin in which late apoptosis up to 23.2% had been reported (Khairi et al., 2014). Early apoptosis was detected in the present study indicating the externalization of negatively charged phosphatidylserine (PS) (Riedl et al., 2011; Jamali et al., 2018), which is mainly localized in the inner layer of the membrane. Late apoptosis is also known as secondary necrosis, causing loss of membrane integrity (Poon et al., 2010).

The cell cycle consists of consecutive four phases that are important in cell division and cell proliferation. The unscheduled proliferation and genomic instability give rise to the accumulation of tumor cells (Hanahan and Weinberg, 2000). The finding in this study suggests that NC leaves 80% ethanol extract caused cell cycle arrest at the G0/G1 phase in MCF-7 cells after 72 h of exposure. The results clearly showed a significant increase of cell distribution in the G0/G1 phase accompanied by a decrease of cell distribution in phase S. Accumulation of cells in the G0/G1 phase has been reported in MCF-7 cancer cells (Sun and Hai Liu, 2006; Choi and Kim, 2008) and other cancer cell lines (Fan et al., 2015; Karade and Jadhav, 2018) after plant extract treatment. A similar finding was also reported in a study in which epirubicin caused the G0/G1 cell cycle arrest on MCF-7 and T47D cells (Xiong et al., 2016). Upon detecting the DNA damage, cell cycle checkpoints stop the progression of the cell cycle from one phase to the next phase to ensure the fidelity of genetic information (Vermeulen et al., 2003). The arrest of MCF-7 cells at the G0/G1 phase prevents the cells from entering the next phases of the cell cycle, the S and G2/M phases, which eventually inhibits the synthesis of DNA and suppresses proliferation of the cells. To find out the possible mechanism of the extract to induce apoptosis and cell cycle arrest in MCF-7 cells, the qPCR assay was performed. The upregulation of cytochrome c links with the upregulation of Bax expression and downregulation of Bcl-2 (Ilhan, 2020). Bax executes its action on mitochondria by promoting the release of cytochrome c, *via* increasing the permeability of mitochondria membrane, into the intracellular space (Kilbride and Prehn, 2013). The inhibition of *Bcl-2* activity involves with the heterodimer formation on *Bcl-2* by Bax gene to counteract the antiapoptotic effects of *Bcl-2* (Adams and Cory, 2007). The liberation of cytochrome c would activate apoptosome, which then activates caspase-9 and the effector caspases, caspase-3 and caspase-7 leading to cell death (Olsson & Zhivotovsky, 2011; Westphal et al., 2011; Looi et al., 2013). The morphological and biochemical changes are associated with caspase activation upon apoptotic stimuli (Duclos et al., 2017). The upregulation of caspase-9 and caspase-7 expressions was detected upon NC treatment on MCF-7 cells. Activation of the effector caspases *via* proteolytic cleavage by caspase-9 and cytochrome c is believed to play a crucial role in the execution of apoptosis (Hassan et al., 2014). These results might suggest that NC extract exerts apoptosis through the intrinsic pathway. The expression of *p21* (tumor suppressor gene) stops the cell cycle progression from dividing damaged cells. The binding of p21 on CDKs or cyclin-CDK complexes disrupts these interactions and inhibits cell cycle progression (Karimian et al., 2016; Kreis et al., 2019). Upregulated *p21* and downregulated *CDK2* support the G0/G1 cell cycle arrest in the previous data. Interestingly, cyclin *E* in the present study was found to be upregulated, which suggested that cyclin *E* independently binds to *CDK2* for halting the cell cycle. It was suggested that inhibition of breast cancer cell MCF-7 proliferation may be linked to the upregulation of cyclin E and p21 as well as downregulation of *CDK2* upon NC extract treatment. In addition, p21 was found to be associated with apoptosis. A study found the cytochrome c blockage and caspase-3 inactivation in HCT116 *p21*−/− but not in HCT *p53*−/− and HCT116 Bax−/− of colon cancer cells after treatment with curcumin. The study also found a reduced expression of *Apaf-1* in the HCT116 *p21*−/− cells, whereas it was not expressed in wild type (Gogada et al., 2011). Cytochrome c release and Apaf-1 are required for caspase-9 activation of the intrinsic apoptosis pathway (Zhou et al., 2015), which in turn activates executioner caspases, including caspase-3 and caspase-7. Based on the present findings, it is suggested that *p21* upregulated expressions may have been involved in apoptosis activities by promoting the cytochrome c release to inhibit the proliferation of MCF-7 cells upon treatment.

## Conclusion

The present research data suggest that NC leaves ethanol extract inhibits cellular growth in MCF-7 cells by inducing apoptosis and cell cycle arrest. As supported by the flow cytometric analysis, upregulation of Bax, cytochrome c, and caspases (caspase-9 and -7) with downregulation of Bcl-2 in MCF-7 cells suggest that NC leaves ethanol extract promotes apoptosis *via* the intrinsic pathway. In addition, upregulation of p21 and cyclin E, and downregulation of CDK2 expressions suggest that cell cycle arrest occurs in MCF-7 cells upon NC leaves’ ethanol extract treatment. Through this finding, the upregulation of cytochrome c may be associated with the upregulation of p21, which ultimately leads to apoptosis induction. Other than that, upregulation of cyclin E alone may have caused cell cycle arrest, in which overexpression may reduce the proliferation of MCF-7 cells. The GCMS analysis revealed that D-pinitol, myo-inositol, hexadecanoic acid (palmitic acid), octadecanoic acid (stearic acid), oleic acid, and phenol, 2,4-bis(1,1-dimethylethyl) compounds may have contributed to the aforementioned anticancer effects of the NC leaves ethanol extract. Nevertheless, other compounds that could not be identified in the GCMS library might have also played some roles in the anticancer effects of the NC leaves ethanol extract. Therefore, further research is still required to identify other possible bioactive compounds possessing anticancer effects and evaluate all the possible bioactive anticancer candidates. Additionally, studies involving an *in vivo* model are still necessary to further analyze NC anticancer effects and the mechanism of action.

## Data Availability

The raw data supporting the conclusions of this article will be made available by the authors, without undue reservation.
