# Supplementary material for: An In Vitro Anticancer Activity Evaluation of Neolamarckia cadamba (Roxb.) Bosser Leaves’ Extract and its Metabolite Profile
Source: Front Pharmacol. 2021 Oct 13;12:741683. doi: 10.3389/fphar.2021.741683 (PMC8548635; doi:10.3389/fphar.2021.741683)
Supplement: Supplementary file 1 [file DataSheet1.docx]

**Supplementary information**

**An *in vitro* Anticancer Activity Evaluation of *Neolamarckia cadamba* ([Roxb.](https://en.wikipedia.org/wiki/Roxb.)) [Bosser](https://en.wikipedia.org/wiki/Jean_Marie_Bosser)** **Leaves Extract and its Metabolite Profile**

***Shakirah Razali^1^, Al’aina Yuhainis Firus Khan^1^, Alfi Khatib^2,3*^, Qamar Uddin Ahmed^2^, Ridhwan Abdul Wahab^1^ and Zainul Amiruddin Zakaria^4,5^****

^1^ Department of Biomedical Science, Kulliyyah of Allied Health Sciences, International Islamic University Malaysia, 25200 Kuantan, Pahang DM, Malaysia; [shakirahrazali@gmail.com](mailto:shakirahrazali@gmail.com); [alainayuhainis@gmail.com](mailto:alainayuhainis@gmail.com); [ridhwan@iium.edu.my](mailto:ridhwan@iium.edu.my)

^2^ Pharmacognosy research Group, Department of Pharmaceutical Chemistry, Kulliyyah of Pharmacy, International Islamic University Malaysia, 25200 Kuantan, Pahang DM, Malaysia; [alfikhatib@iium.edu.my](mailto:alfikhatib@iium.edu.my); [quahmed@iium.edu.my](mailto:quahmed@iium.edu.my)

^3^ Faculty of Pharmacy, Airlangga University, Surabaya 60155, Indonesia

^4^ Laboratory of Halal Science Research, Halal Products Research Institute, Universiti Putra Malaysia, Serdang, Malaysia; zaz@upm.edu.my, [drzazakaria@gmail.com](mailto:drzazakaria@gmail.com)

^5^ Department of Biomedical Science, Faculty of Medicine and Health Sciences, Universiti Putra Malaysia, Serdang, Malaysia

*****Correspondence: Alfi Khatib: [alfikhatib@iium.edu.my](mailto:alfikhatib@iium.edu.my); Zainul Amiruddin Zakaria: zaz@upm.edu.my, [drzazakaria@gmail.com](mailto:drzazakaria@gmail.com)

**Appendix A**

Table S1. List of genes related with apoptosis and cell cycle arrest used, respectively.

Figure S1: The GCMS chromatogram of *N. cadamba* ethanol extract

Table S1.

| Gene | Forward (5ʹ-3ʹ) sequence | Reverse (5ʹ-3ʹ) sequence |
| --- | --- | --- |
| *B-actin* | CGGCGCCCTATAAAACCCA | ATCATCCATGGTGAGCTGGC |
| *GAPDH* | GACAGTCAGCCGCATCTTCT | GCGCCCAATACGACCAAATC |
| *Bax* | GAACCATCATGGGCTGGACAT | ATGGTCACGGTCCAACCACC |
| *Bcl-2* | ATGTGTGTGGAGAGCGTCAA | GGGCCGTACAGTTCCACAAA |
| *Cytochrome c* | CCCAAGAAGTACATCCCTGGAAC | GGCAGTGGCCAATTATTACTCA |
| *Caspase-7* | GGAGAAAGCTCATGGCTGTGT | TCCCCTTGGCTGTGTTTTG |
| *Caspase-9* | TGACCCCAGAATTGACCCTG | AAGGATTCGCTCTTGCGTC |
| *Cyclin-E* | CGGCCTATATATTGGGTTGGC | GGCTGCTGCTTAGCTTGTAAAC |
| *CDK2* | GCATCTTTGCTGAGATGGTGACTC | TAGAAGTAACTCCTGGCCACACC |
| *p21* | GTGGCTATTTTGTCCTTGGGC | TTCTGACATGGCGCCTGAA |
|  |  |  |


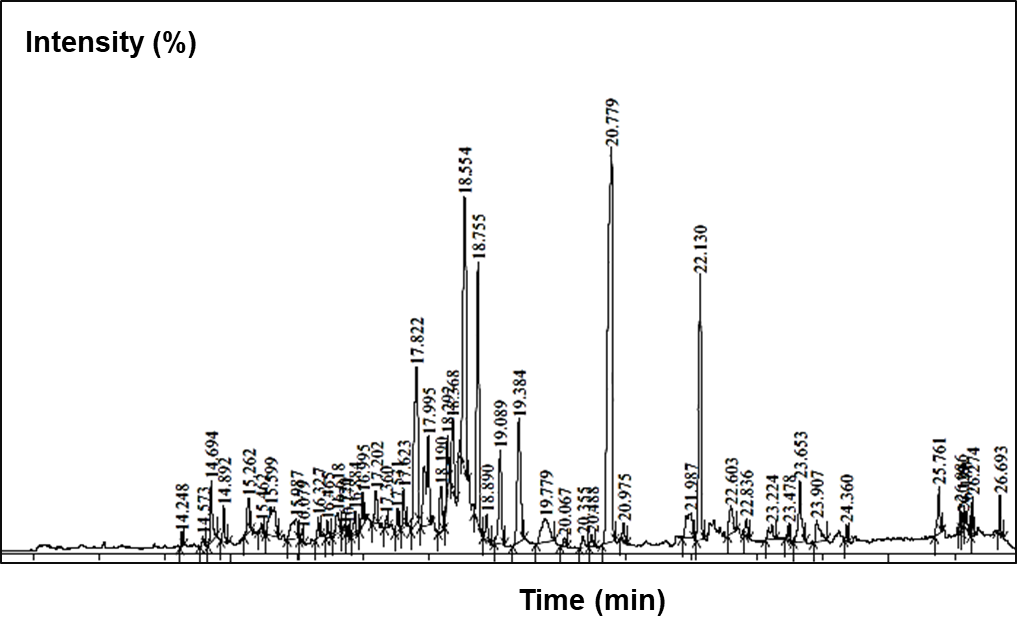


Figure S1.
